# Exploration of multifaceted molecular mechanism of angiotensin-converting enzyme 2 (ACE2) in pathogenesis of various diseases

**DOI:** 10.1016/j.heliyon.2023.e15644

**Published:** 2023-04-20

**Authors:** Aditi D. Kunvariya, Shivani A. Dave, Zeal J. Modi, Paresh K. Patel, Sneha R. Sagar

**Affiliations:** a Department of Pharmaceutical Chemistry, L.J. Institute of Pharmacy, L J University, Ahmedabad 382 210, India

**Keywords:** Angiotensin-converting enzyme 2, Angiotensin II, Renin-angiotensin system, SARS-CoV-2, Cancer, Neurodegenerative diseases

## Abstract

Angiotensin converting enzyme 2 (ACE2) is a homolog of ACE (a transmembrane bound dipeptidyl peptidase enzyme). ACE2 converts angiotensinogen to the heptapeptide angiotensin-(1–7). ACE2 and its product, angiotensin-(1–7), have counteracting effects against the adverse actions of other members of renin-angiotensin system (RAS). ACE2 and its principal product, angiotensin-(1–7), were considered an under recognized arm of the RAS. The COVID-19 pandemic brought to light this arm of RAS with special focus on ACE2. Membrane bound ACE2 serves as a receptor for SARS-CoV-2 viral entry through spike proteins. Apart from that, ACE2 is also involved in the pathogenesis of various other diseases like cardiovascular disease, cancer, respiratory diseases, neurodegenerative diseases and infertility. The present review focuses on the molecular mechanism of ACE2 in neurodegenerative diseases, cancer, cardiovascular disease, infertility and respiratory diseases, including SARS-CoV-2. This review summarizes unveiled roles of ACE2 in the pathogenesis of various diseases which further provides intriguing possibilities for the use of ACE2 activators and RAS modulating agents for various diseases.

## Graphical abstract

(The widely known components of RAS include angiotensinogen, renin, angiotensin (ANG) I and II, and angiotensin-converting enzyme (ACE). Angiotensin II exerts its biological actions via angiotensin II type-1 receptors (AT1R) and angiotensin II type-2 receptors (AT2R). ACE2 is a homolog of ACE, and it uses ANG II as a key substrate to produce ANG-(1–7), via the Mas receptor (MasR). Angiotensin II and ANG-(1–7) are involved in the pathogenesis of various diseases like hypertension, neurodegenerative diseases, respiratory diseases, SARS-CoV-2, Cancer and Infertility. ACE2 functions in the opposite way as ACE and counterbalances the activity of other RAS system. Therefore, ACE2 represents new therapeutic targets for many diseases.)

**Figure:**
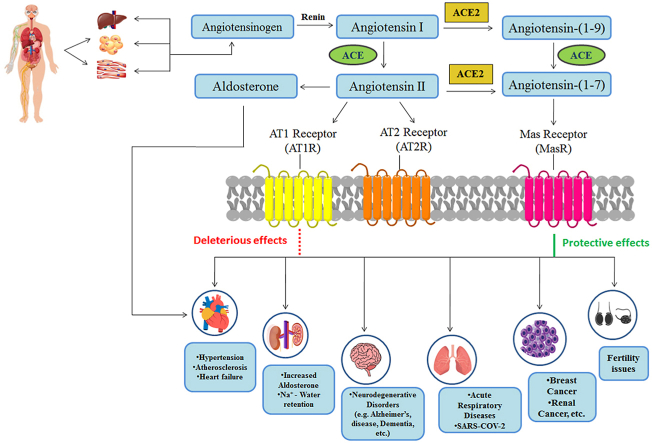

## Abbreviations

ACE: Angiotensin-converting enzyme
ACE2: Angiotensin-converting enzyme 2
RAS: Renin-angiotensin system
ANG: Angiotensin
AD: Alzheimer's disease
PD: Parkinson's disease
HD: Huntington's disease
AT1R: Angiotensin II receptor type 1
AT2R: Angiotensin II receptor type 2
ACEI: Angiotensin-converting enzyme inhibitor
ARB: Angiotensin receptor blocker
VEGF: Vascular Endothelial growth factor
MasR: Mas1 oncogene receptor
NF-ĸB: Nuclear factor Kappa B

## 1 Introduction

The renin-angiotensin system (RAS) is considered as an organized hormonal cascade that has an essential role in governing numerous physiological functions in many organs and it is much more complex than previously thought [1,2]. The RAS is persistently developing with the identification of new components, functions, drugs and subsystems. Till date, many components of RAS, including angiotensin II (ANG II), ANG III, ANG IV, and angiotensin-(1–7) [ANG-(1–7)] have been identified, but out of them, ANG II and ANG-(1–7) have attracted more attention among research communities due to their significant involvement in different diseases together with COVID-19 [3,4]. ACE2 has two domains: the amino-terminal catalytic domain and the carboxy-terminal domain. The catalytic domain consists of the zinc metallo-peptidase domain which has active sites for many substrates and inhibitors [5]. Angiotensin-converting enzyme (ACE) is the vital enzyme involved in the formation of bioactive ANG II, which is an endogenous potent vasoconstrictor responsible for the pathogenesis of hypertension [6,7]. Various ACE inhibitors (ACEI) like captopril, enalapril, lisinopril etc. have proven their efficacy and are used clinically as successful antihypertensive agents [8]. An ACE enzyme and its isoforms have attracted attention in recent pandemic and life threatening conditions caused by SARS-CoV-2, which is accountable for millions of deaths. The SARS-CoV-2 virus is transmitted by the ACE2 receptor and RAS of the host works as a vehicle to enter in human cells followed by viral replication [9,10]. Viral binding to ACE2 causes detaching of ACE2 and results in accumulation of ANG II and thereby affecting the occurrence of vasoconstriction, fibrosis, arrhythmogenesis, hypertrophy, proliferation, oxidative stress, and cardiac dysfunction [11]. On the other hand, there is an association between ACE2 and some cardiac comorbidities such as heart failure, secondary hypertension, coronary artery disease, and cardiomyopathies, which may lead to COVID-19 defenselessness and severity [12].

ACE-2 has been shown to convert Aβ_43_ (a highly amyloidogenic form of Aβ responsible for plaque formation) to Aβ_42_, which in turn is cleaved by ACE1 to Aβ_40_ which has reduced toxicity. A lower level of ACE2 promotes the early deposition of Aβ_43_ and leads to the development of Alzheimer's disease [13,14]. The physiological level of ANG II peptide has cholinergic inhibitory activity, which may increase the risk of white matter hyper-intensities, and Aβ induced neurotoxicity [15]. In addition, the central activity of ANG II is involved in the damage of dopaminergic degeneration in the substantia nigra region which is accountable for the progression of Parkinson's disease (PD) [[16], [17], [18]]. The different isoenzymes and receptors of ACE (ACE1, ACE2 and ACE3) are also involved in male fertile health, mainly steroidogenesis, epididymal contractility, and sperm cell function, while in female, RAS plays a significant role in reproductive events, particularly follicle development, granulosa-lutein (GL) cell apoptosis, ovulation, etc. [[19], [20], [21]].

A prominent cause of morbidity in current times is cancer. ANG II has been proven as a growth factor and stimulates tumour neovascularization, a vital requisite for tumour growth [22,23]. Several cytokines like interleukin-6 (IL-6), IL-12, IL-8, monocyte chemoattractant protein-1 (MSC-1), activator protein-1 (AP-1) and reactive oxygen species (ROS) involved in inflammation and tumour progression are elevated and upregulated by AT1R [24]. The clinical data shows that use of ACEI and angiotensin receptor blockers (ARBs) reduces tumour growth, tumour associated angiogenesis, and inhibits metastasis [25]. There is a reduction in breast and lung cancer in hypertensive patients who have used ACEIs for a long time. ACEIs also reduce the risk of oesophageal, prostate and renal cancer [26]. In contrast to that, ACE2/ANG-(1–7) has shown to be down regulated in breast cancer, lung cancer and pancreatic cancer.

Due to the active involvement of ACE2 enzyme in various pathological events and conditions, especially in SARS-CoV-2, ACE2 has become one of the most emerging targets in the new drug discovery for SARS-CoV-2. An effective activation of ACE2 by a drug may prevent the occurrence of various cardiac events, which is one of the reasons for morbidity and mortality in the patients who have recovered from SARS-CoV-2. Therefore, there is an urgent need to discover a potent and bioavailable ACE2 activator that may be used in SARS-CoV-2 infection and other physiological conditions [12].

This review focuses on the molecular mechanisms of ACE2 in various diseases like cancer, respiratory disease, cardiovascular disease, neurodegenerative disease and infertility. The role of ACE2 in other physiological conditions like diabetes, stroke, etc. is not included in this work. Moreover, this review covers the molecular mechanisms of ACE2 enzyme only. Other isoforms of ACE enzyme are not described in this review.

Scientific articles from the years 1990–2023 were taken from the electronic databases, i.e., Pubmed, Google Scholar, Science Direct, Web of Science and Scopus. The terms used for this review included ACE2, ANG-(1–7), ACE2 activators, RAS, Role of ACE2 in respiratory disease and SARS-CoV-2, Role of ACE2 in cancer, Role of ACE2 in neurodegenerative diseases and Role of ACE2 in infertility.

### 1.1 Renin-angiotensin system (RAS)

The renin-angiotensin system plays a crucial role in maintaining blood pressure and electrolyte homeostasis. The RAS cascade begins with the release of renin from the juxtaglomerular cells. Renin converts angiotensinogen to the decapeptide ANG I. Further, ACE catalyses the hydrolysis of ANG I and converts it into an octapeptide ANG II. ANG II has been considered as the main bioactive component of RAS since many years. ANG II functions mainly by binding to G protein-coupled receptors namely AT1R and AT2R. Now a days, many other RAS components have been identified with similar, distinct or opposite effects compared to ANG II. ANG-(1–12), ANG III, and ANG A exert similar effects as ANG II. Other RAS component peptides like ANG-(1–7), ANG-(1–9), alamandine and ANG IV exert protective effects opposite to ANG II. Overall, the RAS is constituted by two opposite arms: one is ANG II/AT1R, which mediates oxidative, pro-inflammatory, hypertensive, profibrotic, vasoconstrictor and proliferative activities. Another arm is composed of ANG-(1–7)/MasR, which exhibits anti-oxidant, anti-inflammatory, anti-fibrotic, vasodilatory and anti-proliferative activities [27,28].

### 1.2 ACE2/MasR/ANG-(1–7)

An octapeptide ANGII is converted to the heptapeptide ANG-(1–7) by ACE2. ACE2 also converts ANG I to ANG-(1–9), which is then cleaved by either neprilysin (NEP) or ACE to yield ANG-(1–7) [29]. ANG-(1–7) exerts its actions via activation of Mas receptor. Mas receptor is widely distributed throughout all tissues; it is highly expressed in the brain and testes and minimally expressed in the heart, kidneys, lungs, vasculature, adipose tissue, and skeletal muscle. ACE2 produces ANG-(1–7) but limits the production of ANGII. ACE2 is a multifaceted enzyme implicated in the regulation of cardiovascular, renal, lung and cerebral functions [27,29].

### 1.3 ACE2 in cardiovascular diseases

RAS plays a crucial role in preserving healthy cardiovascular (CV) functions and is linked to a number of diseases and conditions that affect the heart, including hypertension, coronary heart disease, myocarditis, thrombosis and congestive heart failure. A role for the RAS in the preservation of heart function and cardiac hypertrophy was suggested by the pharmacological inhibition of ACE or ANG II receptors. Variations in platelet function and aggregation appear to have an interaction with RAS, which is correlated to a higher risk of thrombosis [30,31].

Activation of ANG II is linked to the prothrombotic condition brought on by high blood pressure. By interacting with AT1R, AT2R and AT4R, ANG II directly activates cells. It can also indirectly stimulate cells by producing endothelin-1 and/or bradykinin, which subsequently connect with their own receptors to mediate cell activation. Despite the evidence that AT1R on platelets mediates ANG II-induced platelet aggregation *in vitro*, this receptor population does not seem to be involved in the development of microvascular thrombus. The beginning of ANG II-enhanced microvascular thrombosis appears to be mediated by AT2R activation, whereas AT4R activation is involved in the stabilisation phase (cessation of blood flow) of the thrombosis response. The thrombosis in response to ANG II is unaffected by genetic AT1R deficiencies or pharmaceutical antagonists of these receptors. The start and flow cessation responses to chronic ANG II infusion are largely prevented by selective inhibition of either endothelin 1 or bradykinin 1 receptors. According to the clinical data, arteriole thrombosis is increased in ANG II-induced hypertension [30,31].

Role of ACE2 in CV diseases is well documented in previous reports [[32], [33], [34], [35]]. Cardiac overexpression of ACE2 exerts a protective effect on the heart during myocardial infarction. Lack of ACE2 increases vascular inflammation in apolipoprotein E knockout mice, who are more susceptible to atherosclerosis, and the inflammatory response promotes the development of atherosclerotic plaque. The degree of left ventricular dilatation and the inhibition of unfavourable left ventricular remodeling were associated with the upregulation of the ACE2 gene in the left ventricular myocardium of patients with severe heart failure [30,31]. Brain ACE2 works in concert with the other RAS molecules (ACE, ANG II, and AT1R) to protect autonomic and baroreflex function, release nitric oxide, reduce oxidative stress, and prevent the onset of attenuated hypertension. Given its potential therapeutic effects, ACE2 may be used to treat heart failure, hypertension, and other cardiovascular diseases. Exogenous ACE2 administration or increasing brain ACE2 through endogenous ACE2 activity stimulation may have positive effects in certain pathologic conditions and represent a new strategy for the development of novel therapeutic agents in the future [36,37]. The use of ACEI/ARB may be able to reduce CTRCD (Cancer Therapy-Related Cardiac Dysfunction) after chemotherapy with anthracyclines and/or trastuzumab, according to the findings of some meta-analyses [38].

The pathogenesis of numerous cardiovascular disorders, including myocarditis, heart failure, atherosclerosis and many others, is significantly influenced by pro-inflammatory cytokines. An immediate and constant decrease in disease severity is observed in a wide range of inflammatory disorders when IL-1 activity is effectively blocked. Interleukin-1 (IL-1) is a molecule that is interconnected with both acute and chronic inflammation as well as other related diseases including cancer and cardiomyopathy. When compared to non-cancer patients, individuals with melanoma, colon, lung, head, neck, or breast cancer have higher levels of IL-1, which activates pathways for tumourigenesis and resistance to radiation and chemotherapy. A significant element in the survival, invasiveness, and therapy resistance of cancer is inflammation in the tumour microenvironment, which is mediated by IL-1. IL-1 is directly produced by cancer cells, and they also encourage other cells to emit it.

As among IL-1 family members, IL- 1β has been shown to be a potential treatment for a variety of auto-inflammatory illnesses, including psoriasis, acute gout, and rheumatoid arthritis. The management of atherosclerosis, heart failure, myocarditis, and doxorubicin-induced cardiotoxicity was effective because of the suppression of IL-1. The IL-1 receptor antagonist anakinra blocks the IL-1 receptor, which lowers the action of IL- 1β, and is one of three IL-1 blockers that have been recognized. Additionally, monoclonal anti-interleukin- 1β antibody canakinumab and the soluble decoy receptor rilonacept can be used.

Several solid tumours have been found to contain elevated levels of IL-1, and individuals with IL-1β-producing tumours have a negative prognosis. Recent cardiovascular studies have shown the IL-1 blocking drug canakinumab to have positive outcomes. As anticipated, canakinumab considerably decreased cardiovascular disease and death rates in people with high mortality risks [39,40].

### 1.4 ACE2 in respiratory diseases

The blood capillaries in the lungs are one of the primary sites of ACE expression and ANG II production in the human body. Recent studies have demonstrated that, along with pulmonary fibrosis and acute lung illness ACE2 plays a significant role in lung pathophysiology [41]. It has been demonstrated that ANG II, rather than AT1R, directly induces the creation or development of pulmonary artery smooth muscle cells in *in vitro* cell culture models [29]. Activation of endogenous ACE2 has hypothesized to change the balance of the RAS from the vasoconstrictive, proliferative axis (ACE-ANG II-AT1R) to the vasoprotective axis [ACE 2-ANG-(1–7)-Mas], resulting in the prevention of pulmonary hypertension (PH). The most common causes of PH are chronic obstructive pulmonary disease (COPD), left heart failure, schistosomiasis, exposure to high altitudes, medications, chemicals, and HIV infection. These risk factors have been predicted to cause an imbalance between vasoconstrictor and vasodilator processes when they are combined with genetic predisposition variables [41]. A recent study has been demonstrated that the human airway epithelial cell gene ACE2 is induced by interferon, which could help explain why SARS-CoV-2 and other respiratory illnesses are becoming more common [[41], [42], [43]]. The variable presence of ACE2, the receptor that SARS-CoV-2 uses for host insertion, is thought to be the cause of the greater likelihood of co-infection among COVID-19 (coronavirus illness 2019) patients [44]. The down regulation of ACE2 caused by the SARS spike protein appears to make lung failure worse [45]. In patients with an upper respiratory infection (URI), analysis of the appearance of ACE2 in the pharyngeal epithelium was carried out, and its relationships with clinical traits and serological data were explored. URI patients (n = 125) had significantly greater levels of ACE2 gene expression than healthy controls (HC) people (n = 52). Age and body temperature were significantly and favourably correlated with the expression level of the ACE2 gene, which may help explain the rising co-infections with SARS-CoV-2 and other respiratory diseases [44]. GSK2586881, a recombinant form of human angiotensin-converting enzyme 2 (rhACE2) can lessen the likelihood of acute lung injury [[46], [47], [48], [49]].

On chromosome 17q23, the human ACE gene (dcp1) has a restriction fragment length polymorphism in intron sixteen's coding sequence that is indicated depending on whether a 287-bp repeat is present (insertion, I) or absent (deletion, D). ACE activity is increased by the human ACE2 D allele and this polymorphism dictates function [45]. The ACE I/D allele polymorphism and mortality in ARDS (Acute Respiratory Distress Syndrome) cohorts have been linked in a number of studies and meta-analyses [42,45,46]. Some people who take ACEI experience a dry, ineffective cough [47]. The inflammatory and immunological systems use the ACE for a variety of purposes. In addition to converting ANG I to ANG II, ACE also breaks down substance P and bradykinin, two protussive peptides. The increased cough reflex and suppression of aspiration may be caused by the impaired metabolism of these peptides caused by ACE inhibition [48].

#### 1.4.1 Role of ACE2 in SARS-CoV-2

The SARS-CoV-2 virus attacks lung alveoli and spike protein of the virus binds to ACE2 receptors. SARS-CoV-2 replicates using host ribosomal machinery and the RNA-dependent RNA polymerase (RdRp) enzyme and produces new copies of +ssRNA and its polyproteins. After that, SARS-CoV-2 mediates inflammatory events by destroying the infected pneumocytes of alveolar sac. This causes increased capillary permeability of endothelial cells and vasodilation of blood vessels. Although lungs are the primary site of infection, other organs and tissues can also be affected, leading to a wide variety of clinical conditions. Prognosis of SARS-CoV-2 is favorable in many patients, but it may lead to critical illness, respiratory distress, shock, thromboembolism, multi organ failure, and eventually death in some patients. Many patients with chronic underlying diseases have a poor prognosis as in the case of patients with cardiovascular disease, patients with a high ratio of CD4^+^ T cells to CD8^+^ T cells and elderly individuals [50].

ACE2 is present in the membranes of cells via an active site. However, for the results of a few proteolytic enzymes such as ADAM 17 (A disintegrin and metalloprotease 17) which scrape the ACE2 protein groups from the surface of the cell, they can be cleaved and supplied in the circulation. ACE2 shedding can be up-regulated by ANG II but usually circulates in imperceptible amounts. Likewise, ACE2 shedding is higher in pathological conditions such as heart disorders, diabetes, hypertension and obesity [51]. Considering specific ACE2 expression in RAS & COVID-19, freely circulating ACE2 is segregated from membrane bound ACE2. Kidney, gut, heart, testicles and lungs have membrane ACE2 as demonstrated widely. The utilitarian role of circulating ACE2 in the lungs seems to be clinically inapplicable under physiological conditions due to stunted levels [52,53]. Envelope (E), matrix (M) and spike (S), as well as nucleocapsid (N) proteins 3–5 are the structural proteins of SARS-CoV-2. S1 and S2 are subunits of S protein that are responsible for cell binding and penetration [54]. Binding to the cellular receptors is done by coronaviruses using the homotrimeric spike conjugated protein (comprising the S1 and S2 subunits in every spike monomer) on the envelope. The interaction between SARS-CoV-2 spike macromolecule and ACE2 cell receptor is a vital step for SARS-CoV-2 to enter target cells [[55], [56], [57], [58], [59], [60]].

Virus internalization is initiated by the binding of the spike protein of SARS-CoV-2 with ACE2 receptors [61]. ACE2 which is attached to the human host cell wall has the binding of S1 domain. It is identical to SARS-COV-2 and this complex is transferred to a cell [54,62]. Viral genome is released into the cytoplasm by cleavage of the S2 domain. Here the viral genome is changed into replicase polyproteins that regulate RNA replication and synthesis. Intracellular mechanisms are used to prepare non-structural and structural proteins of viruses. Golgi-endoplasmic reticulum intermediate compartment sees the budding of these proteins in it. Different target cells are then infected by the fabrication and liberation of novel viral particles [62].

It is observed that type 2 alveolar cells in the lung and also cells in the heart, blood vessels, GIT, kidney, nasal, and oral mucosa experience type 1 transmembrane protein with carboxy-mono peptidase activity. SARS-CoV-2 infection due to the incorporation of virus into the host cell leads to a decrease in *in vivo* expression of ACE2 [54]. SARS-CoV-2 enters the host cell and down regulation of ACE2 takes place. Because of decreased ACE2 levels prior to infection, it is particularly troublesome in individuals suffering from various medical conditions like diabetes, heart disorders, etc. The ACE:ACE2 ratio is then escalated by SARS-CoV-2 infection, thus advancing constriction in blood vessels. Patients with myalgic encephalomyelitis/chronic fatigue syndrome (ME/CFS) are the same as these patients have a greater risk for COVID-19 because of a significant decrease in ACE2 expression [63].

The SARS-CoV-2 protein identifies human ACE2 with a greater binding affinity than the spike of SARS-CoV. Preclinical studies showed that down regulation of ACE2 expression is caused by ACE2 activating RAS, after attachment of SARS-CoV to its receptor, resulting in exorbitant synthesis of ANG II [53]. SARS-CoV infection is completely restrained by the absence of ACE2 expression. Specifically, ACE2 has greater interaction with the receptor binding domain (RBD) of the SARS-CoV-2, in comparison to other virus infections from the same family. By an increase in ACE2 expression, virus capacity to penetrate the cells is significantly intensified [52].

Increased ACE2 level is related to the dominant allele ‘D’ of ACE I/D polymorphism. In the Asian populations, a notable positive correlation was seen between the frequency of D allele and the number of SARS-CoV-2 infected cases. Decreased ACE2 levels could be presumed in subjects harbouring the major allele D of ACE I/D polymorphism as ACE2 and ACE levels are contradictory to each other. Moreover, SARS-CoV-2 is counter indicated by the soluble form of ACE2 protein which is rarely present in the circulation. The regulation of the invasion of the host cell up to substantial levels is done by the high probability of SARS-CoV-2 and ACE2 receptor interactions [61].

Above all, the virus binds to ACE2 receptors with a high affinity through a particular S macromolecule region called RBD. The virus is stopped by S-RBD antibodies from coming into and infecting a cell by stopping or neutralizing its biological result. However, it's not known if these responses are related to protection against later infections [60].

**Fig. 1:**
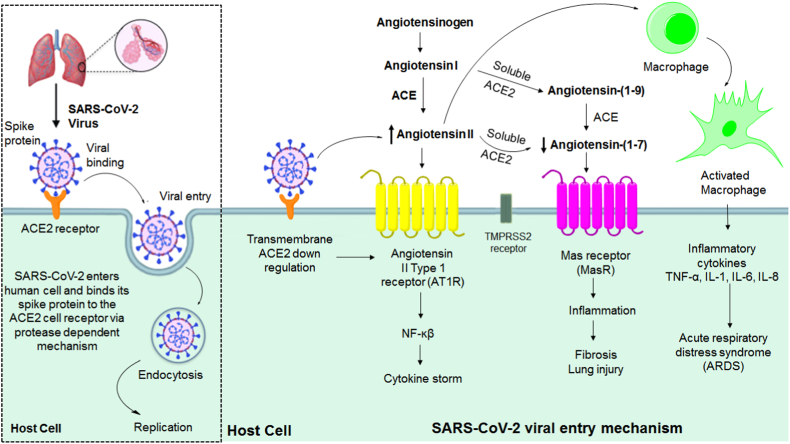
Renin-angiotensin system (RAS) targeting SARS-CoV-2 viral entry mechanism. (The process of SARS-CoV-2 entering host cells in the lungs is represented here. SARS-CoV-2 enters the lungs via spike glycoprotein of the virus. Spike protein binds to ACE2 on cells and allows viral entry. Transmembrane protease serine 2 (TMPRSS2) also participates in this process. Viral entry to the host cell further produces mature virion which causes immunological reactions and inflammatory responses. ANG II contributes to the key events of the inflammatory response. It also plays a role in the recruitment of infiltrating inflammatory cells into tissues, either directly activating them or by controlling the expression of adhesion molecules and chemokines by resident cells. Other pro-inflammatory effects of ANG II are also possible. ANG II stimulates MCP-1 (Monocyte chemoattractant protein-1), a crucial component of the inflammatory process. Nuclear factor Kappa B (NF-ĸB) is activated as a result of AT1R activation, and this in turn triggers the production of a number of cytokines and adhesion molecules, including TNF-α, IL-6, IL-8, MCP-1, and transforming growth factor (TGF)-β. In addition, ROS and cytokines like TNF-β may activate NF-ĸB, which increases cytokine production, the creation of a positive feedback loop, and thereafter amplification of the inflammatory process leading to lung injury.).

Overall, the down regulation of ACE2 disturbs the balance between RAS and ACE2/ANG-(1–7)/MasR axis. In animal studies, it has been found that ACEIs (e.g., Captopril, Enalapril) can decrease plasma ANG II levels and increase ANG-(1–7) and ACE2 expression. Moreover, ARBs (e.g. Losartan, Telmisartan, etc.) increases plasma levels of ANG II, ANG-(1–7) and ACE2. Recombinant ACE2 can also increase ACE2 level in COVID-19 patients. The stimulation or overexpression of ACE2 is associated with a decrease in inflammation, lung damage, edema, and the onset of ARDS as shown in Fig. 1. Use of these repurposed drugs is an effective strategy to reactivate the down regulated ACE2 in COVID-19 patients. These drugs can be given alone or in combination as prophylactics or therapeutics against the SARS-CoV-2 virus [[64], [65], [66], [67], [68]].

##### 1.4.1.1 Role of ACE2 in SARS-CoV-2 and other comorbid conditions

ACE2's utility in SARS-CoV-2 infection induces a severe inflammatory response and life-threatening symptoms. Low ACE2 activity was observed in many cancer patients when compared to healthy individuals. ACE2 has anti-tumour roles in many cancers. ACE2 level is also correlated with a low epithelial to mesenchymal transition (EMT) score in various respiratory cancer cell lines. In lung cancer patients, lower expression of ACE2 is responsible for up regulation of Zinc Finger E-Box Binding Homeobox 1 (ZEB1). ZEB1 further promotes EMT induction and this shifts cells to a more mesenchymal phenotype. ACE2 down regulation negatively correlates with tumour infiltration and prognosis in KIRP (kidney renal papillary cell carcinoma) and UCEC (uterine corpus endometrial carcinoma). Low ACE2 expression in cancer patients with COVID-19 plays a critical role in promoting tumour phenotypes that further aggravate the disease [69,70].

SARS-CoV-2 affects pneumocytes and myocardiocytes leading to severe injuries to lung and heart tissues. ACE2 down regulation causes vasoconstriction, endothelial damage, activation of the coagulation system and clot formation with enhanced thrombosis. These conditions aggravate severe hypoxemia, alveolar damage, ARDS, myocardial ischemia and heart damage. Patients with myocardial inflammation, interstitial edema and cardiac fibrosis have abnormal regulation of the intracellular Ca^++^ and K^+^ concentrations leading to arrhythmias and subsequently heart failure. Overall systemic damage may also lead to multi organ failure [71].

##### 1.4.1.2 Role of ACE2 and COVID-19 associated musculoskeletal manifestation

All the RAS components are found in the skeletal muscle. ANG II binds to AT1R and causes an increase in ROS production, a decrease in protein synthesis, protein degradation and development of fibrosis. Therefore, activation of the classical RAS pathway and ANG II show detrimental consequences in skeletal muscle mainly fibrosis, insulin resistance and muscular atrophy. An increase in the circulating ANG II also reduces muscle protein synthesis by decreasing the levels of insulin-like growth factor type 1 (IGF1) and altering the protein kinase B (Akt)/mammalian target of rapamycin (mTOR) signaling pathway. On the other hand, ANG-(1–7) produces opposite effects on skeletal muscles. In murine models, ANG-(1–7)/MasR axis can increase the muscle strength and functionality of animals by promoting the synthesis of muscle proteins. In SARS-CoV-2 infected patients, dysregulation of RAS and hyper-inflammatory events could induce skeletal muscle atrophy. It is reported that 50% of intensive care unit (ICU) patients had muscle wasting mainly of diaphragm and lower limb muscles. These complications might remain for years after hospital discharge [[72], [73], [74]].

### 1.5 Role of ACE2 in cancer

Even though cancer survival rates are increasing, it continues to be a leading cause of death globally [75]. Colorectal cancer (CRC), one of the most frequently diagnosed fatalities, is a significant factor in the global burden of cancer-related mortality. According to growing evidence, the RAS may contribute to the growth of tumours, primarily through angiogenesis. Therefore, medications that block the RAS may have anti-tumour effects [76]. Another important global public health issue is renal cancer. The most prevalent type of renal cancer is renal cell carcinoma (RCC), which develops from the renal epithelium. One of the main analeptic options for the treatment of progressive RCC is the use of macrophages and interleukins. Lung cancer is also the leading cause of cancer mortality, particularly in western nations [76,77].

The active hormone, ANG II, functions by binding to transmembrane receptors. In order to increase inflammation and leukocyte adhesion, activation of the AT1R stimulates the production of endothelial adhesion molecules like intercellular adhesion molecule 1 (ICAM-1) and vascular cell adhesion molecule 1 (VCAM-1). Both the AT1R and the AT2R increase VEGF (vascular endothelial growth factor) intensity levels. Patients receiving chemotherapy for a progressive solid tumour appear to have a genuine longer overall survival when taking ACEI/ARB [77].

RAS inhibitors (ACEIs and selective ARBs) are a subclass of medications that have been linked to improved cancer patient outcomes and a decline in the incidence of the disease. ANG II promotes tumour neovascularization, a crucial prerequisite for tumour growth. Their uses may be connected to a decline in the renal cancer outcomes. The administration of VEGFR-inhibitors (vascular endothelial growth factor) as well as ANG II receptor activation upregulates VEGF and inhibits tumour growth. Anti-EGF-receptor (EGFR) drug is widely used approach in cancer therapy [[76], [77], [78]].

**Fig. 2:**
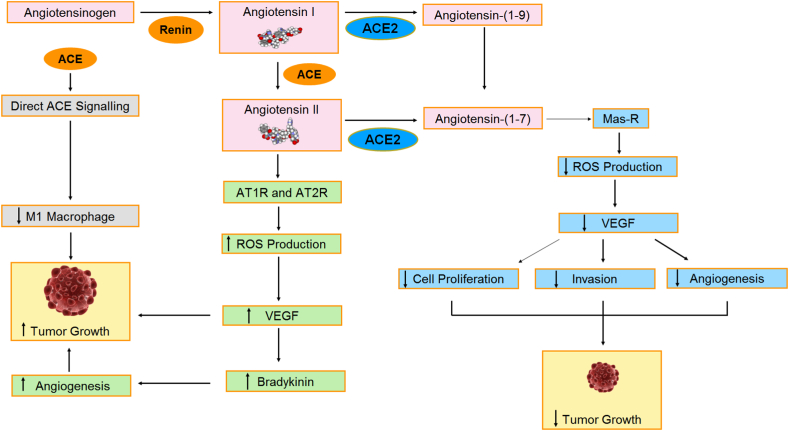
Role of members of the RAS system in Cancer. (ANG II, a member of the RAS system acts as a potent mitogen. ANG II is involved in cell proliferation, inflammation, migration and angiogenesis process. ANG II increases ROS production which further induces VEGF production and causes cancer. Effect of ACE2 enzyme in cancer is complicated. It may have both positive and negative roles in cancer therapies. It can decrease cancer cell growth, invasion and angiogenesis in breast cancer, lung cancer, colon cancer and pancreatic cancer but it promotes the migration and invasion of human renal cell carcinoma cells.)

ACE2 inhibits metastasis, angiogenesis, invasion and cell growth in several cancers like breast cancer, colon cancer, lung cancer and pancreatic cancer (Fig. 2). Apart from these effects, ACE2 also promotes migration and invasion in renal cancer via the Mas-mediated AKT (serine-threonine kinase) signalling pathway. The ACE2/ANG-(1–7)/MasR axis reduces the production of VEGF in drug-resistant tumours which ultimately inhibits angiogenesis and tumour growth. Therefore, ACE2/ANG-(1–7)/MasR might emerge as new therapeutic targets for the treatment of cancer [78,79].

### 1.6 Role of ACE2 in neurodegenerative diseases

**Fig. 3:**
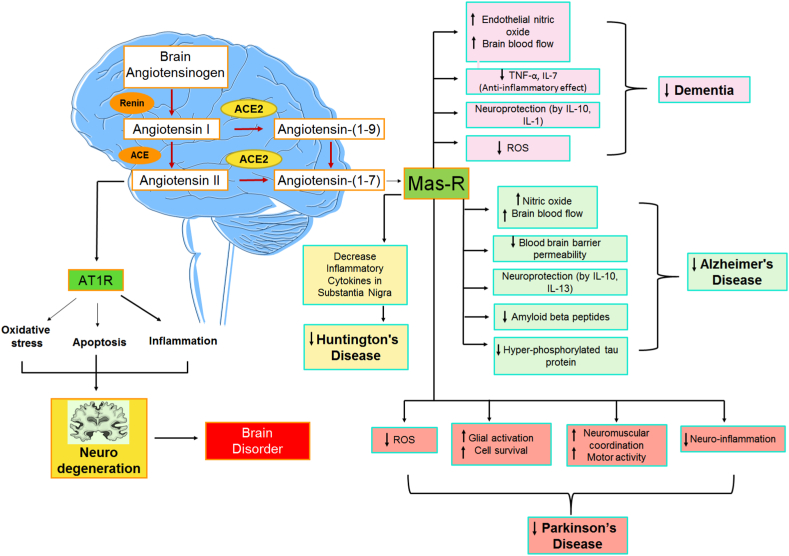
Role of RAS in various neurodegenerative diseases. (A decreased level of ACE2 is responsible for several neurodegenerative diseases. A decrease in ACE2/ANG-(1–7)/MasR causes an increase in neuroinflammation, oxidative stress, cognitive impairment, β–peptide formation and tau phosphorylation. Therefore, ACE2/ANG-(1–7)/MasR axis components lead to neuroprotection in CNS disorders like AD, PD and HD.).

RAS is involved in neurodegenerative diseases like Parkinson's disease (PD), stroke, and Alzheimer's disease (AD) (Fig. 3). The ACE, ANG II and AT1R axis are majorly responsible for oxidative stress, apoptosis and neuroinflammation leading to neurodegeneration in various brain diseases. ANG II increases oxidative stress and produces pro-inflammatory genes and other chemicals that may harm the endothelium and brain tissue. These pro-inflammatory mechanisms begin with the activation of NF-ĸB and NAD(P)H oxidase, and intra mitochondrial oxidative stress may be a key player in this process [[80], [81], [82]].

#### 1.6.1 Alzheimer's disease

The pathogenesis of AD occurs via the change of neuronal proteins, like amyloid precursor protein (APP) and Aβ peptide which form amyloid plaques in the brain cortex and hippocampus regions [83]. ANG II mediates many of the anti-cholinergic, pro-inflammatory and vasopressor actions of RAS through AT1R.

ACE2 is expressed in endothelial and arterial smooth muscle cells, and non-vascular cells like astroglial cells and neuronal cell bodies. Reduced activity of the ACE2/ANG-(1–7)/Mas axis is strongly linked to over activity of ACE/ANG II/AT1R and with AD related pathology. AD pathology is directly related to ACE activity. The visible differences in enzyme activity were more proficient in the medial hippocampus (44% increase) and para hippocampal hyrax (41% increase) than the caudate nucleus (29%). In the new castle cases, there was a course in the medial hippocampus and caudate nucleus leading to more ACE activity related to neuropathological change. Conversion of ANG I to ANG II enhances the secretion of acetylcholine from the cerebral cortex and creates a modulatory effect on the rate of acetylcholine in the brain [84]. By the increased generation of ANG II, aged Tg-2576 AD mice could increase symptoms of AD by activating the AT1R. Significantly, genetic deficiency of the ANG II/AT1R in a transgenic AD model, resulted in decreased Abeta plaque formation [85]. In immune histological studies, AT1R was localized in the brains of aged Tg2576 AD mice. Immuno histology showed the ANG II-AT1R on hippocampal neurons of 18-month-old Tg2576 mice. The AT1R was localized in close proximity to insoluble Abeta plaque deposits. The increased ACE dependent ANG II generation in the AD brain could increase Abeta generation and symptoms of neurodegeneration by AT1R stimulation. Inhibition of ACE with the brain penetrating, captopril, led to a reduced Abeta plaque load in aged Tg2576 AD mice [86].

#### 1.6.2 Parkinson's disease

Parkinson's disease (PD) is characterized by the abnormal accumulation of α-synuclein which further forms Lewy bodies in the substantia nigra. A recent study demonstrated neuroprotective behavior of ANG-(1–7) by continuous administration of ANG-(1–7) into the right substantia nigra for 4 weeks. It was found that ANG-(1–7) reduced α-synuclein aggregation in the substantia nigra by alleviating autophagy dysfunction in PD [87,88]. Furthermore, ANG-(1–7) stimulates dopamine release in the brain of PD and ACE inhibition leads to an increase in extracellular dopamine. In Parkinson's and Alzheimer's patients, ACE activity is higher and is considered to reflect a response to inflammation of the brain. PD relates to a genetic polymorphism in ACE genes. Animal models demonstrate the role of ANG II in regulating inflammation and damage via AT1R. The central activity of ANG II is also responsible for the damage caused by dopaminergic degeneration in the substantia nigra, caudate nucleus, and putamen by a rise in the levels of nicotinamide adenine dinucleotide phosphate (NADPH), oxidases-derived superoxide, and microglial activation [81,82].

#### 1.6.3 Huntington's disease

Huntington's disease (HD) is a neurodegenerative disorder with a variable-length expansion of the CAG (Cytosine, Adenine, Guanine) trinucleotide repeat in exon 1 of the Huntingtin (HTT) gene. Toxic function of mutant Huntingtin (mHTT) leads to HD pathology. Altered electrophysiological properties in HD contribute to neuronal dysfunction and neurodegeneration. In a recent study, the effects of ANG II and ANG-(1–7) have been investigated using immortalized progenitor striatal cell lines expressing mHTT. ANG II decreased the potassium current in control cells but it had a negligible effect on potassium current in mHTT expressing cells. The reduced effect of ANG II on potassium currents could be due to the decreased expression of AT1R in these cells. On the other hand, ANG-(1–7) decreased the potassium current in mutant cells by activating the Mas receptor. Moreover, ACE activity was shown to be lower in the striatum and substantia nigra of HD patient's brain homogenates. The globus pallidus, caudate, and putamen in the striatal region had decreased enzyme activity when compared to the pars compacta in the substantia nigra. The pars reticulate showed a more pronounced reduction in ACE activity. Additionally, the nucleus accumbens showed a lesser but still noticeable decline in enzymatic activity [89,90].

#### 1.6.4 Diabetic neuropathy

Recent studies show that ANG II activates microglia that produces neurodegeneration due to the release of inflammatory cytokines [85]. In diabetic neuropathy, hyperglycaemic conditions release tissue ANG II by the activation of ACE. ANG II is involved in oxidative stress and endothelial damage that results in vasoconstriction, thrombosis, inflammation and vascular remodeling in epineural vascular tissue.

#### 1.6.5 Schizophrenia

A special attention has been paid to the oligopeptidase known as nuclear distribution element-like 1 (Ndel1) because of its dual role and distinctive functions, such as its ability to form a complex with the schizophrenia (SCZ) susceptible gene product, DISC1 (Disrupted-in-Schizophrenia 1) and to cleave neuropeptides such as neurotensin (NT) and bradykinin (BK), which are important for the response to antipsychotics and for the neuron-generating division of neural progenitor cells, respectively. The formation of Ndel1-DISC1 complex mediates neurite outgrowth and brain formation, and also mediates negatively the Ndel1 oligopeptidase activity. Many studies revealed that Ndel1 expression and enzyme activity is decreased in SCZ patients compared to healthy controls (HCs) paired by age and sex [86,90].

ACEI/ACE2 activators help in the management of neurodegenerative disorders, due to various reasons like, ANG-(1–7) produces neuroprotective action, decrease in pro-nociceptive bradykinin 1 receptors in the peripheral nervous system, activation of Mas receptors releases nitric oxide and prostaglandins that cause vasodilation, bio-synthesis of ANG IV peptide in brain causes enhanced learning and memory functions via activation of AT4R, increase the metabolism of bradykinin and substance P and release of endogenous opioid peptides [83]. Thus, blockage of classic components of the RAS (ARBs and ACEI) or activation of ACE2/ANG-(1–7)/Mas receptor axis components leads to neuroprotection in CNS disorders like AD and PD. Additionally, modulation of the RAS may be an interesting therapeutic strategy for the treatment of HD [81,[91], [92], [93], [94], [95]].

### 1.7 Role of ACE2 in fertility

Alteration in ACE1 and/or ACE2 and/or ACE3 expression is one of the main critical mechanisms underlying infertility in both males and females. Role of the ACE1 gene in male and female infertility has been studied in the past and it was considered as a prospective target that raises the risk factors for infertility. The activation of the RAS in Polycystic Ovary Syndrome (PCOS) suggested a significant relationship between the RAS and PCOS [96]. ACE2 is emerging as a key regulator in the reproductive health of males and females. The effect on reproduction is mediated by ANG-(1–7) [[97], [98], [99], [100], [101]]. Various pathways (ACE1/ANG II/AT1R, ACE1/ANG II/AT2R and ACE2/ANG-(1–7)/Mas) involved in female reproductive events must have correct balance for follicle development, ovulation and granulosa-lutein (GL) cell apoptosis [97,[102], [103], [104], [105]]. ACE1/ANG II/AT1R and ACE2/ANG-(1–7)/MasR pathways are also involved in male fertile health particularly for steroidogenesis, sperm cell function and epididymal contractility [73,[106], [107], [108]].

ACE2 is present in human and rat ovaries and its end products, ANG-(1–7) peptides are also located in ovarian compartments and follicular fluid [101]. Gonadotropin affects the ovarian expression of ACE2, ANG-(1–7) and MasR. This data demonstrated the role of ACE2 in ovarian physiology [105]. In the male reproductive system, ACE2 is selectively expressed by adult Leydig cells in the testis. ACE2, ANG-(1–7) and MasR have also been detected in the testis and are mainly located in the Leydig cells [100]. Previous studies have suggested the importance of ACE2 in the male reproductive system. Severe spermatogenesis impairment is due to the lower levels of ACE2, ANG-(1–7), and MasR compared to fertile subjects. This evidence proved the key role of ACE2 in the regulation of spermatogenesis [100]. Several controversial results were obtained for the use of ACEIs as effective drugs for the management of infertile men with idiopathic oligospermia with respect to sperm quality and quantity. However, some studies suggested beneficial effects of ACEIs on sperm quantity and quality by blocking the conversion of bradykinin into inactive peptides [98]. Overall, ACE2 may serve as a novel therapeutic target for the treatment of male and female infertility after validation through extensive research.

## 2 Conclusion

ACE2 is much more than just a receptor for SARS-CoV-2 as it is extensively expressed in various tissues and organs including lung, heart, kidney, brain, testis, gut and many others. This comprehensive review comprises the evidence in support of a critical role for ACE2 in the pathogenesis of many diseases including cardiovascular disease, respiratory disease, SARS-CoV-2, cancer, neurodegenerative diseases and infertility. The ACE2/ANG-(1–7)/MasR axis exerts anti-inflammatory, antioxidant, vasodilation, anti-fibrosis, and anti-apoptosis actions opposite to those of the ACE/ANG II/AT1R axis. Based on these findings, agents targeting ACE2/ANG-(1–7)/MasR may emerge as a breakthrough treatment strategy for complex diseases along with comorbid conditions. Understanding the molecular mechanism of RAS and its components may bring forth new opportunities in developing therapeutic agents such as ACE2 activators, ARBs, ACEI and recombinant ACE2 to balance the ACE2/ANG-(1–7)/MasR and ACE/ANG II/AT1R arms of the RAS.

## Author contribution statement

All authors listed have significantly contributed to the development and the writing of this article.

## Data availability statement

This review article was prepared using already available reported references.

## Funding sources

No funding source has been utilized in this work.

## Author contribution

ADK, SAD & ZJM carried out literature search and compilation of data. PKP & SRS prepared and revised manuscript.
